# Oxidative Stress and MicroRNAs in Vascular Diseases

**DOI:** 10.3390/ijms140917319

**Published:** 2013-08-22

**Authors:** Alessandra Magenta, Simona Greco, Carlo Gaetano, Fabio Martelli

**Affiliations:** 1 Istituto Dermopatico del’Immacolata-IRCCS, Vascular Pathology Laboratory, Via dei Monti di Creta 104, Rome 00167, Italy; E-Mail: ale.magenta@gmail.com; 2 Policlinico San Donato-IRCCS, Molecular Cardiology Laboratory, Via Morandi 30, San Donato Milanese, Milan 20097, Italy; E-Mail: greco.simona@gmail.com; 3 Division of Cardiovascular Epigenetics, Department of Cardiology, Internal Medicine Clinic III, Goethe University, Theodor-Stern-Kai 7, Frankfurt am Main 60590, Germany; E-Mail: gaetano@em.uni-frankfurt.de

**Keywords:** microRNAs, oxidative stress, vascular diseases, mitochondrial dysfunction, endothelial dysfunction

## Abstract

Oxidative stress has been demonstrated to play a causal role in different vascular diseases, such as hypertension, diabetic vasculopathy, hypercholesterolemia and atherosclerosis. Indeed, increased reactive oxygen species (ROS) production is known to impair endothelial and vascular smooth muscle cell functions, contributing to the development of cardiovascular diseases. MicroRNAs (miRNAs) are non-coding RNA molecules that modulate the stability and/or the translational efficiency of target messenger RNAs. They have been shown to be modulated in most biological processes, including in cellular responses to redox imbalance. In particular, miR-200 family members play a crucial role in oxidative-stress dependent endothelial dysfunction, as well as in cardiovascular complications of diabetes and obesity. In addition, different miRNAs, such as miR-210, have been demonstrated to play a key role in mitochondrial metabolism, therefore modulating ROS production and sensitivity. In this review, we will discuss miRNAs modulated by ROS or involved in ROS production, and implicated in vascular diseases in which redox imbalance has a pathogenetic role.

## 1. Introduction

### 1.1. MicroRNA Biogenesis and Function

microRNAs (miRNAs) are 21–23 nucleotide long non-coding RNA molecules, that modulate the stability and/or the translational efficiency of target messenger RNAs (mRNA). miRNAs have been demonstrated to be involved in most biological processes, including differentiation, proliferation development, migration and apoptosis (for extensive reviews on miRNA regulation and biogenesis see: [1–3]). Generally miRNAs act as negative regulators of gene expression, albeit few opposite examples have been described [4]. miRNA genes can be expressed as polycistronic transcripts containing multiple miRNAs, as independent transcripts, or can be embedded in introns of protein coding mRNAs.

**Figure 1:**
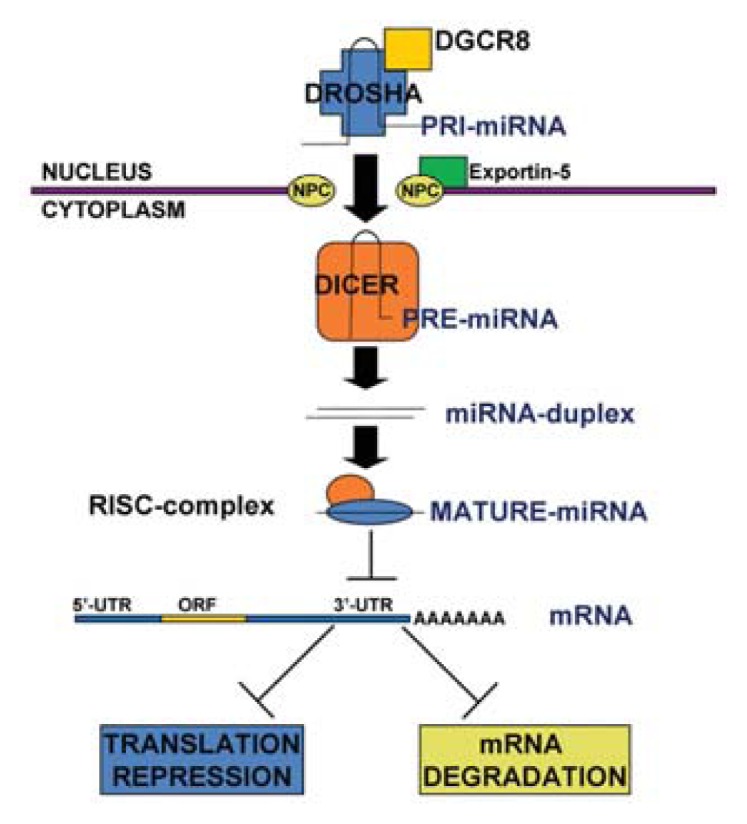
miRNA biogenesis. The pri-miRNA is cleaved to generate a 70–100 nucleotide long hairpin-shaped pre-miRNA by the complex Drosha/DGCR8, in the nucleus. The pre-miRNA is shuttled to the cytoplasm by Exportin 5 and then processed by the ribonuclease III Dicer, to form the mature 22-nt miRNA:miRNA* duplex. Afterwards, one strand of the duplex, the mature single-stranded miRNA, is incorporated into the RISC complex.

miRNA biogenesis (Figure 1) begins from a primary transcript, termed the pri-miRNA, a generally thousands nucleotides long mRNA, transcribed by RNA polymerase II; only few miRNAs are transcribed by RNA polymerase III. The pri-miRNA contains the active miRNA in a stem–loop structure. This hairpin undergoes nuclear cleavage by the ribonuclease III Drosha, complexed to the RNA-binding protein DGCR8/Pasha, to generate a 70–100 nucleotides hairpin-shaped pre-miRNA. It is noteworthy that most intronic miRNAs can be processed from unspliced intronic regions before splicing catalysis; however, a subset of intronic miRNAs, named mirtrons, enters in the miRNA-processing pathway without a Drosha-mediated cleavage. Successively, the pre-miRNA is transported to the cytoplasm by the nuclear export factor Exportin 5 (Xpo-5) and further processed by the ribonuclease III Dicer, complexed to TRBP (TAR RNA-binding protein), to form the mature 22-nt miRNA:miRNA* duplex. The complementary strand miRNA* is typically degraded, while the mature single-stranded form is incorporated into the RNA-Induced Silencing Complex (RISC).

Besides few exceptions, metazoan miRNAs base pair imperfectly with their mRNA targets and induce translational inhibition. Mature miRNAs loaded into the RISC mediate translational inhibition of target mRNAs through several different mechanisms. Moreover, RISC-miRNA complexes can shuttle mRNAs to specialized cytoplasmic compartments, the “P-bodies”, that are enriched in mRNA-catabolizing enzymes. Consequently, miRNAs also have an important effect on mRNA degradation. Since each miRNA can target multiple transcripts and individual transcripts may be subject to multiple miRNA regulation, it may prove difficult to find a biological process or function that is not, at least in part, under the influence of miRNAs.

The identification of miRNA targets is crucial to elucidate the role played by each miRNA in a biological function. The rules that guide miRNA/mRNA interactions are complex and still incompletely understood [1].The current paradigm states that a complete pairing between the 3′UTR region of the mRNA target and the “seed sequence” of the miRNA, a region centered on nucleotides 2–7, is required for miRNA-mRNA mediated inhibition. Thus, seed pairing is a necessary requirement for most target prediction algorithms. However, recent studies have demonstrated that also non-canonical miRNA binding can confer target regulation [5]. Specifically, some mRNAs are targeted by miRNAs through recognition of 5′UTR or coding sequences. Moreover, “seedless” miRNA/mRNA interactions have been also demonstrated [6,7].

The complex regulation of targets mRNAs by miRNAs is far from being elucidated, but what is now clear is the importance of the fine-tuning modulation of most biological pathways by miRNAs. Aberrant regulation of miRNA expression, in fact, has been linked with the onset and progression of many conditions, including cardiovascular diseases. A variety of mechanisms has been described underpinning the dysregulation of miRNA level. In most circumstances, the transcription of the miRNA is deregulated. In this respect, recent investigations demonstrated that epigenetic modifications such as histone acetylation or DNA methylation can play an important role [8].

### 1.2. Oxidative Stress and Cardiovascular Diseases

Reactive oxygen species (ROS) is a collective term that includes a number of reactive and partially reduced oxygen (O_2_) metabolites, with some of them being free radicals, such as superoxide anion (O_2_^−^) and hydroxyl radicals (^•^OH), that are extremely reactive molecular species with an unpaired electron in their outer orbital. The third most relevant molecule included in ROS is hydrogen peroxide# (H_2_O_2_), which is more properly a pro-oxidant and non-radical molecule. Formation of ROS is an unavoidable consequence of aerobic metabolism. ROS can be generated within living cells by several sources: mitochondria, plasma membrane NADPH oxidase (NOX) and different enzymes involved in redox reactions such as several oxidases, peroxidase, cytochromes, mono- and di-oxygenases and uncoupled NOS (nitric oxide synthase) [9].

To cope with the burden of oxidative damage, a series of enzymatic and non-enzymatic antioxidant defences have evolved to neutralize ROS [9]. O_2_^−^ may be dismutated by a family of superoxide dismutases (SODs) to H_2_O_2_. Three enzymatic types of SOD have been identified: MnSOD (SOD2) is located in the mitochondria and two isoforms of Cu/ZnSOD are located either intracellularly (SOD1) or extracellularly (ECSOD, SOD3). H_2_O_2_ can be scavenged to water by catalase (CAT) or by glutathione peroxidase (GPx) in presence of reduced glutathione [10]. Glutathione is a non-enzymatic aqueous phase antioxidant, that is the most important and versatile protector from ROS [10].

ROS are highly reactive molecules and, as such, can inflict damage to DNA, proteins, and fatty acids. It is generally believed that redox imbalance caused by excessive ROS production or insufficient scavenging plays a causal role in a wide range of cardiovascular diseases, including systemic and pulmonary hypertension, diabetic vasculopathy, hypercholesterolemia, atherosclerosis, stroke, myocardial infarction and heart failure [11–13]. Moreover, oxidative stress is involved in the pathogenesis of other diseases, such as cancer and pulmonary fibrosis as well as in aging [14].

Physiological ROS levels play an important role in intracellular signaling as second messengers; however, when ROS production is exacerbated or scavenging insufficient, dysregulation of many biological processes occurs. Increased ROS levels, in fact, are known to impair endothelial function in humans, as well as in animal models [15].

Numerous lines of evidence have suggested that the major source of ROS in the vasculature is the membrane associated enzyme complex, NADPH oxidase [16]. NAD(P)H oxidase is a superoxide producing enzyme, present in vascular endothelial, smooth muscle, and adventitial cells that regulates the vascular tone and contributes to the development of atherosclerosis [16]. This oxidase was first described in phagocytes of the immune system where a high level of ROS production is generated within phagocyte vacuoles implicated in host defences, mediating the killing of ingested pathogens [16]. Structurally, the phagocyte NADPH oxidase is composed of two essential membrane-bound components, gp91phox/Nox2 and p22phox, which compose flavocytochrome *b*558, and 4 cytosolic components, p47phox, p67phox, p40phox, and the small G protein Rac1/2 [17].

Four homologues of gp91phox/Nox2, called Nox1 and Nox3 to 5, have been identified in nonphagocytic cells, and the simultaneous presence of multiple Nox proteins was demonstrated in one cell type; however, tissue- and cell-specific variability is exhibited. For example in endothelial and smooth muscle cells the gp91phox subunit to which NADPH and oxygen bind may be substituted with its homologues Nox1, Nox4 or Nox5 [18,19], and this variability may have implications for the action of NADPH oxidase. Nox4 was highly expressed with Nox1 and p22phox and produced superoxide in atherosclerotic lesions, suggesting an important role for Nox4 in their formation. Furthermore, it has been reported that Nox4 expression is decreased by proliferative stimuli, such as serum, platelet-derived growth factor and angiotensin II, in cultured vascular muscle cells (VSMCs). Nox4 expression in endothelial cells (ECs) is significantly increased by serum removal leading to the suppression of cell proliferation and decreased by addition of serum [16]. Therefore, Nox4 plays a pivotal role in the regulation of cell growth and survival in ECs. Along these lines, propionyl-l-carnitine (PLC) that reduces Nox4 activity and consequently ROS production, was shown to increase EC proliferation and to improve post-hindischemic flow recovery and revascularization [20].

Another mechanism by which endothelial function is impaired is the reduction of nitric oxide (NO) bioavailability. Indeed, NO is known to play an important role in vascular tone relaxation, as well as in the inhibition of platelet aggregation, and in the suppression of vascular smooth muscle cell proliferation [15]. Endothelial dysfunction is considered an early feature of both atherosclerosis and vascular diseases in humans, and the grade of endothelial function is a predictor of cardiovascular outcomes [21]. Accordingly, impaired endothelium-dependent vasodilation has been found in the forearm, coronary, and renal vasculature of patients with hypertension, dyslipidemia, diabetes mellitus, and coronary artery diseases [15].

Several molecular mechanisms underpinning endothelial dysfunction and NO bioavailability reduction have been described. In particular, it is important to underline that NOS in specific conditions is also a source of O_2_^−^; *in vitro* studies, in fact, demonstrated that uncoupling of both endothelial and neuronal NOS (eNOS and nNOS respectively) leads to the production of O_2_^−^, rather than NO. Interestingly, the major determinant of NOS uncoupling appears to be availability of the cofactor tetrahydrobiopterin (BH4) [22,23]. ROS such as O_2_^−^ and H_2_O_2_, as well as peroxynitrite (ONOO^−^), another potent oxidant produced by the reaction of O_2_^−^ with NO, induce BH4 degradation that is associated with eNOS downregulation. This, in turn, leads to a reduction of the amount of endothelium-derived NO that is required for vascular relaxation and EC proliferation and survival [15]. Accordingly, it has been demonstrated that BH4 supplementation improves endothelial function *in vitro*. Moreover, in smokers and patients with hypercholesterolemia, hypertension or chronic heart failure, oxidative stress increase correlates with a deficiency of BH4 [15]. In summary, BH4 deficiency and decreased eNOS activity cause endothelial dysfunction through an increase in oxidative stress. Reduced production of NO and increased production of ROS are involved in the impairment of endothelium-dependent vasodilatation in patients with cardiovascular diseases.

Increased oxidative stress levels mediate many pathophysiological processes also in VSMCs [24]. VSMCs form the medial layer of blood vessels and represent a dynamic component of the vasculature. In normal conditions they have a differentiated, contractile phenotype. Upon pathological stimuli, they can undergo hypertrophy or adopt a “de-differentiated” phenotype, producing an excess of extracellular matrix and inflammatory cytokines, dividing and migrating towards the intima. Interestingly, oxidative stress and ROS have been implicated in all of these responses [24].

It must be underlined that cardiovascular diseases are multifactorial, involving a complex interplay between lifestyle (smoking, diet, ethanol consumption, exercise) and fixed (age, genotype, menopausal status, sex) causative factors [11,12]. Endothelial damage often represent the first step in cardiovascular diseases, which exposes these cells and the underlying cell layers to a deleterious inflammatory process, ultimately leading to the formation of atherosclerotic lesions [11–13]. Consequently to lesion formation, production of reactive oxygen and nitrogen species by many cell types including ECs, VSMCs and monocytes/macrophages occurs. Exogenous factors such as smoking and the existence of other disease states such as diabetes, further contribute to oxidative stress production, increasing the risk factors for cardiovascular diseases development.

Given the pivotal role played by oxidative stress in both endothelial and VSMC pathophysiology, we will dissect the molecular mechanisms triggered by redox imbalance, focusing on the role played by microRNAs.

Different miRNAs modulated by oxidative stress involved in endothelial and vascular dysfunction, as well as miRNAs associated to cardiovascular diseases caused by excessive ROS production, such as diabetes or obesity are summarized in Table 1.

## 2. miRNAs Modulated by ROS in ECs and VSMCs

Although a permissible level of free radicals may trigger protective adaptation, a redox imbalance in the vasculature is at the basis of several pathophysiological processes and finally leads to vascular impairment. Indeed, different pathophysiological conditions such as hyperglycemia, hyperlipidemia, hypertension, and aging can all cause and/or exacerbate oxidative stress in ECs. The elevated levels of ROS alter the activity of vascular ECs through different mechanisms at both transcriptional and post-translational levels. In addition, high ROS levels damage mitochondrial DNA and impair mitochondrial biogenesis, which subsequently inhibit the anti-oxidant defense processes.

Different stimuli that produce ROS are known to induce modulation of miRNA expression: UV, H_2_O_2_, ionizing radiation, and anticancer drugs, such as etoposide and anthracyclines [27,28,53]. Numerous studies focusing on miRNA profiling upon oxidative stress exposure have been performed in different tissues, demonstrating the important role played by miRNA modulation in cell response elicited by a redox imbalance. Interestingly, a screening of miRNAs in human fibroblasts exposed to radiation, H_2_O_2_ or etoposide had been performed in order to find common signature in response to genotoxic oxidative stress [27]. Among these, seven miRNAs were found modulated by all treatments, predicting a common signature altered by agents that induce cytotoxicity and oxidative stress [27].

### 2.1. miR-200 Family

A miRNA profiling of Human Umbilical Endothelial Cells (HUVEC) treated for 8 and 24 h with 200 μM H_2_O_2_, shows an upregulation of miR-200 family members [28].

This miRNA family consists of five members, miR-200c and miR-141 clustered on chromosome 12, and miR-200a, miR-200b, and miR-429 clustered on chromosome 1 and it has been widely studied for its role in the epithelial to mesenchymal transition of tumor cells [26]. However, the role of miR-200 family in vascular cell response to oxidative stress displays characteristic features.

In particular, miR-200c and miR-141 are the most up-regulated miRNAs in HUVEC, exposed to H_2_O_2_ for different periods of time, whereas the other three clustered members are up-regulated to a lower extent. Since miR-200c is well expressed in HUVEC, whereas the other family miRNAs are barely detectable, miR-200c is likely the main effector of oxidative stress-induced biological responses in EC. The induction of miR-200c upon H_2_O_2_ was also confirmed in other cell lines, such as human fibroblasts and C2C12 myoblasts and myotubes [28]. Other interventions causing redox imbalance, different from H_2_O_2_, have been tested as well. Specifically, the alkylating agent 1,3-bis(2 chloroethyl)-1-nitrosourea (BCNU), a glutathione reductase inhibitor that blocks the conversion of oxidized to reduced glutathione, is also able to increase miR-200c expression. Moreover, BCNU-induced miR-200c increase is inhibited by the free-radical scavenger *N*-acetyl-l-cysteine (NAC), confirming the oxidative stress dependence of miR-200c upregulation. Interestingly, miR-200c overexpression in HUVEC recapitulates many aspects of the oxidative stress-induced phenotype, since it induces cell growth arrest, apoptosis and cellular senescence. All these effects are mediated, at least in part, by the inhibition of the target ZEB1 by the miR-200 family. Moreover, miR-200 family induction is also present in an *in vivo* animal model of hindlimb ischemia, which is known to generate oxidative stress [32]. Interestingly, in p66^ShcA−/−^ mice, which display lower levels of oxidative stress in basal conditions [14] and after ischemia [32], miR-200c and miR-200b upregulation is markedly inhibited following hindlimb ischemia, further supporting the pivotal role of ROS in miR-200 family induction [28].

**Figure 2:**
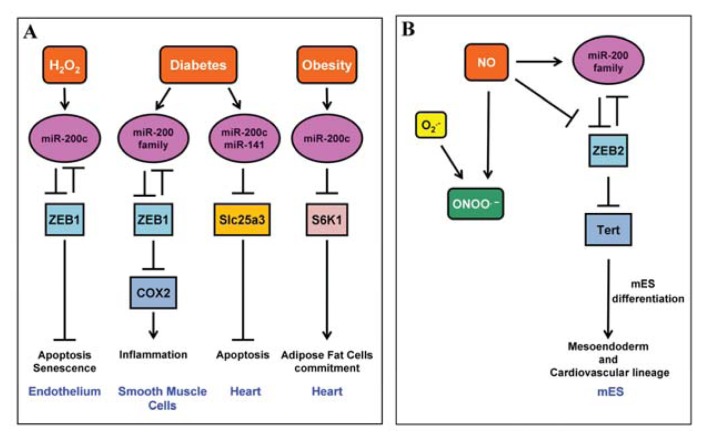
(**A**) miR-200 family role in endothelial dysfunction and in cardiovascular complications linked to diabetes and obesity. This picture summarizes different pathways where a source of ROS or a pathology associated to elevated ROS production (coloured in red) plays a causal role in endothelial or cardiovascular diseases. The tissue or organ district where these mechanisms have been identified are coloured in blue; (**B**) miR-200 family and NO. Schematic representation of the role played by the free radical NO on miR-200 family induction which leads to ZEB2 downmodulation and Tert upregulation, inducing mES differentiation towards the mesendoderm and cardiovascular lineage.

In conclusion, this study first underlined the importance of miR-200 family upregulation, and in particular of miR-200c in EC response to oxidative stress, demonstrating the key role of ZEB1 down-modulation in ROS-induced apoptosis and senescence (Figure 2A).

In agreement with these findings, different studies underline miR-200 family role in apoptosis and senescence also in cancer cells. For example, a pro-apoptotic role of miR-200c has been discovered, showing that this miRNA targets the apoptosis inhibitor FAP1, thereby sensitizing tumor cells to apoptosis [54]. In keeping with a miR-200c role in senescence, it has been shown that miR-200c is upregulated by chronic oxidative stress-induced senescence in human fibroblasts and in human trabecular meshwork cells [55].

Moreover, different reports confirmed miR-200 family upregulation upon oxidative stress. MiR-200c and miR-141 upregulation was, in fact, observed in a cell model of oxidative stress by treatment of House Ear Institute-Organ of Corti 1 (HEI-OC1) cells with different concentrations of tertbutyl hydroperoxide (t-BHP) [56].

Furthermore, miR-200c upregulation upon oxidative stress was also found in a miRNA profiling of mouse primary hippocampal neurons treated with 200 μmol/L H_2_O_2_ for 6 h [25]. Interestingly, miR-200 family is also induced by another important free radical whose regulation plays a pivotal role in endothelial function *i.e.*, NO [57].

Accordingly, NO treatment, miR-200-family over-expression and ZEB2 knock-down all elicit the expression of mesendoderm and early cardiovascular precursor markers, including Flk1 and CXCR4, in mES [57]. Thus, miR-200 family and ZEB2 transcription factor are regulated by NO, indicating a molecular circuitry important for telomerase regulation and early differentiation of mES (Figure 2B).

Finally, a crosstalk between oxidative stress and miR-200 family has been described in other systems as well [58]. miR-200 family induction following H_2_O_2_ exposure has been confirmed in different cell lines *i.e.*, human and mouse immortalized fibroblasts, colon carcinoma (CT26), mammary gland epithelial cells (NMuMG) and human cell lines, melanoma cells (MDA-MB-435S), kidney cells (293T), breast adenocarcinoma (MDA-MB-436 and BT-549) and ovarian adenocarcinoma (SKOV3). Notably, in all these cell lines, all miR-200 family members were upregulated [58].

Another potential mechanism of action is represented by the ability of miR-141 and miR-200a, which display the same seed sequence, to target p38α mitogen-activated protein (MAP) kinase. p38α is a broadly expressed signaling molecule that participates in the regulation of cellular responses to stress [59], as well as in the control of proliferation and survival of many cell types [60]. Indeed, p38α acts as a sensor of oxidative stress [59], and its redox-sensing function is essential in the control of tumor development [61]. Enhanced expression of miR-200 family miRNAs mimics p38α deficiency and increases tumor growth in mouse models, but it also improves the response to chemotherapeutic agents. High-grade human ovarian adenocarcinomas, in fact, are associated with an oxidative stress signature and display high levels of miR-200a and low concentrations of p38α [58]. Since p38α inactivation, pharmacologically or genetically, is known to induce ROS accumulation and activation of antioxidant defenses [59,62], the interaction between miR-200s and oxidative stress response may affect human ovarian carcinogenesis and prognosis. It remains to be determined the relevance of miR-200/p38α axis in vascular cells.

### 2.2. Sirtuin 1-Targeting miRNAs

“Silent mating type information regulation 2 homolog” (sirtuin 1 or SIRT1) is an NAD+-dependent class III histone deacetylase (HDAC) found upregulated under caloric restriction and extending the lifespan of many organisms [63]. SIRT1 activates via deacetylation a myriad of stress-responsive transcription factors (TFs), co-regulators and enzymes, thus playing a key role in metabolic control at cellular, tissue, and whole-body levels [64]. In addition, it shows profound anti-oxidative and anti-inflammatory effects, indicating for SIRT1 an important role in EC biology [65]. A SIRT1 upregulation and/or activation is associated with beneficial effects on ECs, while, excessive ROS or aging, decreases SIRT1 expression leading to endothelial dysfunction [66]. In ECs, SIRT1 activation ameliorates oxidative stress response, prevents endothelial senescence, promotes eNOS-derived NO bioavailability and mitochondrial biogenesis [46,51].

A link between oxidative stress-miRNA-SIRT1 pathways in vascular diseases, and particularly in atherosclerosis and abdominal aortic aneurysm, has been described. Indeed, endothelial senescence is dependent on the age-progressive increase of miR-217, which affects SIRT1 expression, leading to loss of SIRT1 function on its major endothelial targets, Forkhead box protein O1 (FoxO1) and eNOS [47]. Moreover, miR-217 has been found to be negatively correlated with SIRT1 expression in human atherosclerotic plaques and with FOXO1 acetylation status.

Other SIRT1-targeting miRNAs may also play a role in atherogenesis. A SIRT1-targeting miRNA is miR-199, which is downregulated in hypoxic preconditioning, leading to SIRT1 increase and decreased apoptosis in cardiomyocytes [67]. Moreover, miR-199a targets SIRT1 that, in turn, negatively regulates prolyl hydroxylase domain-containing protein 2 (PHD2), required for the de-stabilization of HIF1A.

Bone marrow cells (BMCs) isolated from Acute Myocardial Infarction (AMI) or heart failure showed a significant increase of the pro-apoptotic miRNA miR-34a [68]. Notably, SIRT1 is also a target of miR-34a [44]. In BMCs cultured *in vitro*, blocking of miR-34a inhibited H_2_O_2_-induced cell death, while its overexpression induced cell death and SIRT1 downregulation [68]. Blocking the expression of miR-34a *in ex vivo* pre-incubation of BMCs, increased the effect of the injected BMCs to augment cardiac function in mice after AMI [68].

Interestingly, miR-200a, which is induced by oxidative stress, [28] has been also demonstrated to target SIRT-1 [69], further supporting the idea of a prominent role of miR-200 family members in oxidative stress induced endothelial dysfunction.

### 2.3. miR-21

A miRNA profiling of rat VSMCs treated with 200 μM H_2_O_2_ for 6 hours revealed an upregulation of miR-21 [70]. In this study it was found that miR-21 protects VSMCs from H_2_O_2_-dependent apoptosis and death. In particular, “Programmed cell death 4” (PDCD4) is a direct target of miR-21, involved in miR-21-mediated effects on VSMCs upon oxidative stress. PDCD4, in fact, is a pro-apoptotic protein that fulfils this action inhibiting the activity of the transcription factor AP-1. The latter is a PDCD4 downstream signaling molecule in VSMCs and in other cell types [70,71], which determines life or death cell fates in response to extracellular stimuli, including ROS. In summary, this study shows that miR-21 participates in H_2_O_2_-mediated gene regulation via its target PDCD4 and AP-1 pathway activity [70].

Disturbed flow dynamics lead to the development of atherosclerotic lesions preferentially on sites where the endothelium has become dysfunctional at both cellular and molecular levels [45]. The expression of miR-21 is increased by shear stress and contributes to the protection of ECs by decreasing apoptosis and increasing eNOS and NO production [72]. miR-21 upregulated in atherosclerotic plaques, decreasing the function of superoxide dismutase-2 (SOD-2), a protein important in the mitochondrial oxidative defense, and SPRY-2. This, in turn, leads to ERK/MAP kinase activation, resulting in increased ROS formation and angiogenic progenitor cell (APC) migratory defects [73].

## 3. miRNAs and Mitochondrial Metabolism and Dysfunction

### 3.1. miR-210

Ischemia is characterized by the fall of tissue oxygen tension and by the activation of the hypoxia inducible factor (HIF) family of transcription factors [74,75]. Specifically, under normal oxygen tension, the hydroxylation of HIF1A at two specific proline residues by the “prolyl-4-hydroxylase domain-containing enzymes” (PHDs), targets HIF1A for polyubiquitination and proteosomal degradation by the von Hippel–Lindau tumor suppressor protein (VHL) [76]. Under hypoxic conditions, PHDs are inhibited, saving HIF1A from degradation and allowing it to accumulate.

HIF pathway is responsible for the metabolic shift (“Pasteur effect”) from aerobic mitochondrial oxidative phosphorylation to anaerobic glycolysis. This entails the repression of mitochondrial biogenesis, the induction of most glycolytic enzymes, including lactate dehydrogenase A (LDH-A), which converts pyruvate to lactate [77], and the activation of “pyruvate dehydrogenase kinase” (PDKs) expression. PDK phosphorylates and inactivates the enzyme pyruvate dehydrogenase which converts pyruvate to acetyl-coA, which is then oxidized in the mitochondria in the tricarboxylic acid (TCA) cycle [78].

Mitochondria produce ROS from molecular oxygen by a single electron transport in the respiratory chain. Under normoxia, the low levels of ROS formed during mitochondrial respiration are detoxified by endogenous mechanisms [79]. During ischemia or hypoxia, mitochondrial formation of ROS is increased, causing damage to tissue and cellular compartments [80].

It is often assumed that the switch from oxidative to glycolytic metabolism in cells exposed to hypoxia occurs because oxygen is a limiting factor for cellular respiration and thus for ATP synthesis. However, fibroblasts derived from Hif-1a null mice, which do not switch from oxidative to glycolytic metabolism under hypoxic conditions, display higher ATP levels at 1% O_2_ than wild type (WT) cells have in normoxia, indicating that 1% O_2_ is not limiting for oxidative phosphorylation [81,82]. Conversely, the repression of the oxidative metabolism by HIF is required to maintain redox homeostasis. Under conditions of chronic hypoxia, WT fibroblasts reduce their levels of ROS, while in HIF1A null cells, ROS increase to levels that result in cell death [83]. Thus, oxidative metabolism can be maintained in fibroblasts under hypoxic conditions, but generates toxic levels of ROS.

**Figure 3:**
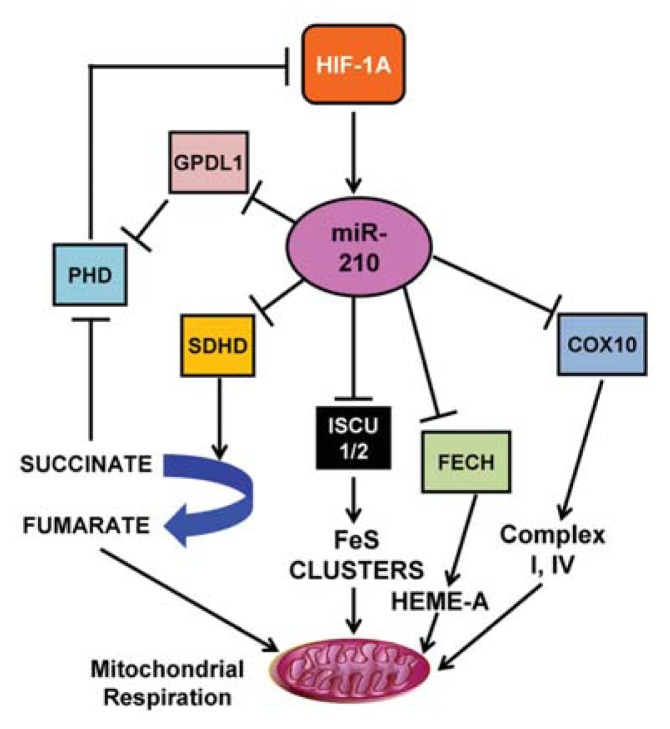
miR-210 and mitochondrial activity regulation. miR-210 inhibits mitochondrial oxidative phosphorylation inhibiting a series of targets: ISCU1 and ISCU2, participating in the assembly of iron sulfur (FeS)clusters, that, in turn, are present in several electron transport chain and TCA cycle components; COX10, a component of mitochondrial complex I and complex IV; FECH, which is the last enzyme in heme biosynthesis; SDHD, one of the subunits of the inner mitochondrial enzyme succinate dehydrogenase that catalyzes the oxidation of succinate to fumarate during mitochondrial respiration. The inhibition of PHDs by succinate and by GPD1L, which feedback to HIF1A, is also shown.

miR-210 is currently regarded as “master miRNA” of hypoxic response, because it was found upregulated by hypoxia in virtually all the cell types tested to date [34,35]. Recent data demonstrate that HIF1A can block both mitochondrial respiration and mitochondrial electron transport chain (ETC) activity, through transcription activation of miR-210 in many cell types [34,35]. miR-210 contributes to this metabolic shift by downregulating several steps of the mitochondrial metabolism, including the ETC complexes (Figure 3). In particular, miR-210 is responsible for the repression of ISCU1 and ISCU2 expression [84,85]. ISCU targeting is particularly important, since ISCU participates in the assembly of iron sulfur clusters, that are present in several ETC and TCA cycle components [37]. Indeed, loss of function of ISCU represses the mitochondrial function and disrupts iron homeostasis [39]. Interestingly, it has been shown that transferrin receptor 1 (TfR) is another relevant target of miR-210 in hypoxia [86]. This may be important for the accurate homeostasis of iron levels within the cell, since miR-210 regulates the assembly of iron-sulfur clusters in hypoxia by means of ISCU repression. In fact, excess iron is toxic since it generates free radicals [38]. Friedreich’s ataxia (FRDA) is a neurodegenerative disease caused by reduced expression of the mitochondrial protein frataxin [87]. The physiopathological consequence of frataxin deficiency is a severe reduction of iron-sulfur cluster biosynthesis, mitochondrial iron overload coupled to cellular iron dysregulation and an increased sensitivity to oxidative stress [87].

At least in part, such overload is mediated by an increase in the expression of the transferrin receptor 1 (TfR), an additional target of miR-210 [86], resulting in a fine-tuning of the iron fluxes through the cellular membrane. Finally, it has been demonstrated that in H9c2 cardiomyocytes, miR-210 targets ferrochelatase (FECH) [40], the last enzyme in heme biosynthesis; this effect is ISCU-independent and more pronounced in normoxia than in hypoxia condition [40]. In view of these effects, miR-210 is able to induce a suppression of ETC function via ISCU, without an associated toxic increase in intracellular iron.

miR-210 not only represses the mitochondrial function disassembling the iron-sulfur clusters by targeting ISCU1/2, but also by targeting important components of the mitochondrial complex I, II and IV [34]. miR-210 negatively regulates “cytochrome c oxidase assembly homolog 10” COX10 [38] and loss of COX10 inhibits the activity of mitochondrial complex I and complex IV [88]. The succinate dehydrogenase complex is the only enzyme that participates in both the TCA cycle and the electron transport chain (complex II). The D subunit of the succinate dehydrogenase complex (SDHD) is also a miR-210 target [41]. SDHD is one of the subunits of the inner mitochondrial enzyme succinate dehydrogenase and catalyzes the oxidation of succinate during mitochondrial respiration. It was hypothesized that miR-210-dependent decrease in SDHD results in increased succinate accumulation, which is a by-product and natural inhibitor of PHD enzymes activity [89].

Another miR-210 mitochondrial target that has been reported is NADH dehydrogenase (ubiquinone) 1 alpha subcomplex 4 (NDUFA4) [90]. Until very recently, this was thought to be an intrinsic part of Complex I, but a very recent work has demonstrated its interaction with Complex IV [91].

An intriguing target that is predicted by several programs and has been experimentally confirmed is glycerol-3-phosphate dehydrogenase 1-like (GPD1-L) [85], which may be involved in the Pasteur effect by modulating NAD^+^/NADH ratios [36]. Although the biochemical function of GPD1L remains to be elucidated, it is highly homologous to glycerol-3-phosphate dehydrogenase (GPD), the catalyst of the glycerol phosphate shuttle, which transfers electrons from cytoplasmic NADH to the mitochondria electron transport chain. Accordingly, also another work indicates that miR-210, via destabilization of GPD1L, contributes to the inactivation of the PHDs, and therefore increases HIF1A expression [92]. This would indicate that miR-210 is both downstream and upstream of HIF signaling.

Targeting mitochondrial complex components, the hypoxia-dependent elevation of miR-210 serves as a potent inhibitor of mitochondrial metabolism. This repression enables the cells to be less sensitive to oxygen for ATP production under hypoxic environment. On the other hand, it may be in conflict with energy demand, if energy-demanding processes, e.g., tissue repair, are required to start. Thus, a prolonged repression of mitochondrial respiration may result in energy starvation, that in turn may be responsible for cell death [34].

Given miR-210 targeting of many mitochondrial components, it is not surprising that manipulation of miR-210 levels leads to mitochondrial dysfunction and oxidative stress.

However, controversial data are reported for miR-210 effects on ROS production under hypoxia. Hypoxia in cancer cells increases the levels of ROS, which can be reversed by blocking miR-210 [93]. Conversely, Chan *et al.* [84] did not observe any change in ROS levels in ECs under hypoxia conditions, but they observed an increase when miR-210 was blocked. It has been found that miR-210 expression in differentiated skeletal muscle cells, neonatal rat cardiomyocytes and in H9c2 cells has a protective effect in response to oxidant stress, reduces mitochondrial ROS production, and decreases mitochondrial mass [33,94].

The discrepancy in ROS accumulation mediated by miR-210 needs further investigations and it may be due to the differences between normal *vs.* cancer cells and also to different methods of ROS detection used among the studies.

While redox imbalance induced by a bolus of H_2_O_2_, does not induce miR-210 expression in ECs [95], this is not necessarily true for all cell systems and/or different forms of oxidative stress. Indeed, ROS generation from diverse sources such as hypoxia, antimycin, rotenone, and platelet-derived growth factor (PDGF)-BB, induces miR-210 expression in adipose-derived stem cells via PDGFR-β, Akt and ERK pathways [96]. Interestingly, there is a positive feed-forward loop between ROS generation and miR-210, since miR-210 itself increases ROS generation by downregulation of ISCU2. Surprisingly, HIF1A is not involved in miR-210 expression in this cell type. Conversely, transcription of miR-210 expression is regulated by NF-κB and Elk1. Finally, miR-210 increases the proliferation and migration of adipose-derived stem cells via ISCU2 and PTPN2 downregulation [96].

Finally, it has been observed that the senescence process in normal human fibroblasts increases the expression of a set of miRNAs including miR-210 [97]. Over-expression of miR-210 induces double-strand DNA breaks and ROS accumulation. Thus, in this case, cellular senescence, normally associated with oxidative stress, is responsible for miR-210 expression. In turn, deregulated miR-210 levels may contribute to sustain oxidative stress and altered cell cycle in the execution of the senescence program in a feed-forward loop manner.

### 3.2. miR-23a/b

Mitochondrial dysfunction is an important mechanism underpinning different human disease states including cardiac diseases, diabetes, and the cardiorenal metabolic syndrome (CRS) [98,99]. Genetic disease is a primary cause for mitochondrial dysfunction and oxidative stress is linked to mitochondrial dysfunction either as a cause, or as a result in pathological states. Mitochondria are major sources of ROS and are also very susceptible to oxidative damage, thereby contributing to mitochondrial dysfunction in a range of diseases.

In response to cardiac hypertrophy both miR-23a and miR-23b are strongly induced [48,100], probably due to a compensatory mechanism to downregulate mitochondrial glutaminase (GLS) [52,101,102]. GLS, in fact, converts glutamine to glutamate that is further catabolized through the TCA cycle, for the production of ATP. Glutamate is also a substrate for glutathione synthesis as well. Proliferating cells utilize glutamine as a major source for energy, nitrogen for biosynthesis, and a carbon substrate for anabolic processes [52,101]. It has been shown that transcriptional regulation of the oncogene Myc coordinates with the expression of genes that promote cells to engage in excessive glutamine catabolism which exceeds the cellular requirement for protein and nucleotide biosynthesis [52]. Myc-dependent glutaminolysis causes a reprogramming of mitochondrial metabolism depending on glutamine catabolism and maintaining cellular viability and TCA cycle anaplerosis. Interestingly, miR-23a/b targets mitochondrial GLS and Myc upregulates glutaminase by down-regulating the expression of miR-23a/b [101,102]. miR-23 was also downregulated in oxidant-induced injury and miR-23 overexpression could rescue H_2_O_2_-induced retinal pigment epithelia cells ARPE-19 cell death and apoptosis [102].

Collectively, this evidence uncovers a role for miR-23 in oxidant-induced injury and mitochondrial metabolism, suggesting a link between Myc regulation of miRNAs, glutamine metabolism, energy and ROS homeostasis. However, the relevance of these mechanisms for vascular cells remains to be elucidated.

### 3.3. miRNAs Involved in Mitochondrial ATP Level Regulation

It has been shown that in the failing heart there is a structural alteration of mitochondria, as well as an increased number and a reduced size [49]. Moreover, mitochondrial injury correlates with ventricular dysfunction indexes, such as ejection fraction and end-diastolic pressure and with the degree of orthosympathetic stimulation in congestive heart failure [98]. In addition, during the transition from left ventricular hypertrophy to failure there is a reduction of mitochondrial protein involved in ADP rephosphorylation [103].

In an interesting study, it was determined that specific miRNAs are able to affect mitochondrial integrity in cardiomyocytes [104]. These miRNAs are miR-15b, miR-16, miR-195 (belonging to miR-15 family), and miR-424 which all share the same seed sequence and are regulators of ATP levels, downregulating the expression of their common protein target: the ADP-ribosylation factor-like 2 (Arl2) mRNA. Knockdown of Arl2 resulted in ATP levels reduction and degeneration of mitochondria.

Considering that mir-15 family is upregulated in different heart diseases (reviewed in [105]), such as ischemia-reperfusion injury in mice and pigs [105,106] or cardiac hypertrophy [100], a consequent mitochondrial degeneration and ATP levels reduction occurs in these pathological conditions.

While the relevance of these pathways for vascular cells remains to be elucidated, it is worth noting that miR-15 and miR-16 impair human circulating pro-angiogenic cell functions and are increased in pro-angiogenic cells and serum of patients with critical limb ischemia [107].

## 4. miRNAs in Vascular Diseases Associated to Obesity and Diabetes

Oxidative stress and inflammation are involved in the initiation and progression of diabetes and obesity, two strictly related conditions [108,109].

Obese individuals experience elevated morbidity and mortality from nearly all forms of cardiovascular diseases. Increased adiposity, in fact, leads to inflammatory and elevated ROS production that both represent risk factors for cardiovascular diseases [110].

Diabetes is also associated with accelerated atherosclerotic disease affecting arteries that supply the heart, brain, and lower extremities [111]. Moreover, diabetic cardiomyopathy is a major diabetic complication; diabetes and impaired glucose tolerance increase cardiovascular disease risk 3- to 8-fold [111]. Finally, new blood vessel growth in response to ischemia is impaired in diabetes, resulting in decreased collateral vessel formation in ischemic hearts and in non-healing foot ulcers [112].

Several lines of evidence indicate that hyperglycemia causes tissue damage by a single upstream event: mitochondrial overproduction of ROS [50,113,114]. In the diabetic microvasculature, this is a consequence of intracellular hyperglycemia. In the diabetic macrovascular and in the heart, in contrast, this appears to be a consequence of increased oxidation of fatty acids, resulting in part from pathway specific insulin resistance. Thus, several groups are trying to unravel the role of miRNAs in metabolic and vascular complications, which are the leading cause for morbidity and mortality in diabetic and obese patients [114].

### 4.1. miRNAs in Obesity-Associated Systemic Inflammation

One of the emerging obesity-associated vasculopathy and cardiovascular diseases risk factors is chronic low-grade inflammation. In obese subjects, there is an increased oxidative stress in adipose tissue associated with adipose tissue dysfunction, characterized by impaired adipocyte maturation and production of pro-inflammatory adipocytokines by dysfunctional adipocytes, as well as increased infiltration of macrophages into the adipose tissues where they produce inflammatory chemokines [110]. Moreover, adipose tissue dysfunction is often associated with endoplasmic reticulum and mitochondrial oxidative stress production [45].

A fundamental cytoprotective effect on cardiovascular diseases is played by heme oxygenase-1 (HO-1) that displays antioxidant, anti-inflammatory, and anti-apoptotic effects. Interestingly, HO-1 activity in obese mice is lower than in controls and a sustained increase in HO-1 protein levels was shown to ameliorate insulin resistance and compensatory hyperinsulinemia [43].

Three miRNAs have been shown to target HO-1, *i.e.*, miR-155, miR-183, and miR-872 and to contribute to the production of proinflammatory adipocytokines, oxidative damage, and apoptosis [43]. In obese rat adipocytes miR-221 and miR-222 are upregulated and they are both known to induce hypoxia by inhibiting endothelial cell migration, proliferation, and angiogenesis [115]. These two miRNAs, in fact, are highly expressed in VSMCs and ECs and their roles have been extensively studied in vascular cell physiology [116,117]. In particular, they have been shown to have pro-proliferative, pro-migration, and anti-apoptotic effects in VSMCs; in contrast, miR-221/222 play antiproliferative, anti-migration, and pro-apoptotic roles in ECs [116]. The opposite effects between the two cell types might be related to the different expression profiles of their target genes, p27(Kip1), p57(kip2) tumor suppressors, and c-Kit. p27(Kip1) and p57(Kip2) are highly expressed in VSMCs, but not in ECs. In contrast, c-Kit is highly expressed in ECs, but not in VSMCs. It has been shown that p27(Kip1) and p57(Kip2) have an anti-proliferative effect on VSMCs and ECs, in contrast, c-Kit is a pro-proliferative gene in these vascular cells. Thus, different availability of these target gene mRNAs in VSMCs and ECs might be related to, at least in part, the different cellular effects of miR-221/222 in these two cell types [116].

Moreover, c-Kit targeting by these two miRNAs in ECs affects the angiogenic properties of c-Kit ligand stem cell factor, inhibiting EC ability to form new capillaries [117]. It has been also reported that miR-221 and miR-222 overexpression reduces the expression of the endothelial nitric oxide synthase (eNOS) in Dicer siRNA-transfected cells [31]. As previously described impaired NO bioavailability plays a causative role in endothelial dysfunction and is a hallmark of patients with atherosclerosis and cardiovascular diseases.

Impaired or delayed growth of ECs and enhanced growth of VSMCs are crucial cellular events in the pathogenesis of atherosclerosis, therefore a miR-221/222 dysregulation represents a critical event in vascular disease pathogenesis.

Finally, in adipose tissue of obese mice, miR-221/222-induced hypoxia is responsible for inducing inflammatory adipocytokines and for increasing miR-27 [118]. This miRNA is associated with impaired adipogenesis through the inhibition of its targets PPARgamma and C/EBPalpha, two key regulators of adipogenesis, thus contributing to adipose tissue dysfunction and obesity-associated vasculopathy [115].

### 4.2. miRNAs and VSMC Dysfunction in Diabetes

Inflammatory responses in VSMCs play a key role in the development of atherosclerosis [119]. In diabetic conditions, an increase of pro-inflammatory mediators such as advanced glycation end products (RAGE), cyclooxygenase-2 (COX-2), cytokines, such as, interleukin-6 (IL-6), tumor necrosis factor-a (TNF-a), and chemokines such as monocyte chemoattractant protein-1 (MCP1) and Fractalkine (CX3CL1), has been extensively reported [42,114]. All these inflammatory mediators promote proliferation, oxidative stress, and monocyte binding to VSMCs leading to VSMC dysfunction and consequently to vascular complications associated with diabetes [120].

Interestingly, a recent study shows the upregulation of miR-200 family members in VSMCs of diabetic mice (db/db) compared to control db/+ mice [121]. In particular, the authors found that miR-200b, miR-200c and miR-429 expression levels are increased, while the protein levels of their common target ZEB1 are decreased in VSMCs of diabetic mice. Overexpression of miR-200 mimics, in fact, downregulating the transcriptional repressor ZEB1, leads to the transcription of the inflammatory genes cyclooxygenase-2 (COX-2) and monocyte chemoattractant protein-1, promoting monocyte binding to VSMCs, therefore eliciting a proinflammatory response. In keeping with these data, ZEB1 occupancy at inflammatory gene promoters is reduced in db/db VSMCs [121]. The authors conclude that the disruption of the reciprocal negative regulatory loop between miR-200 and ZEB1 elicits pro-inflammatory responses of VSMCs, exacerbating the vascular complications in diabetic conditions (Figure 2A).

Epigenetic mechanisms, including post-translational modifications of histones have been shown to be implicated in the upregulation of inflammatory genes in ECs and VSMCs under diabetic conditions [29,114,122]. VSMCs from diabetic mice, in fact, show a decreased promoter occupancy of the histone H3 lysine-9 methyltransferase Suv39h1 and consequently of the associated repressive epigenetic mark histone H3 lysine-9 trimethylation (H3K9me3) of inflammatory genes [29].

In an interesting study, miR-125b is found significantly upregulated in VSMCs of diabetic mice (db/db) compared to control mice db/+, and is demonstrated to direct target Suv39h1, leading to enhanced expression of inflammatory genes [123]. These data suggest that reversal of transcription repression could be a key mechanism for the enhanced expression of inflammatory genes in diabetes.

Interestingly, miR-125b is also induced by oxidative stress in human keratinocytes HaCaT exposed to H_2_O_2_ [124], further supporting the idea that epigenetic changes elicited under diabetic conditions, at least in part, pass through oxidative stress-dependent modification of miRNA expression.

### 4.3. miR-200 Family

In conditions of over-nutrition/obesity, increases in mitochondrial biogenesis, potential and mitochondrial DNA content, have been reported [30]. Accordingly, ROS levels are raised, whereas glutathione is depleted and the redox state becomes more oxidized.

In keeping with these findings, Zucker obese (ZO) rats, a genetic model for obesity and hypertension, display more mitochondria in the heart and cardiac dysfunction compared to controls Zucker lean (ZL) [30].

Interestingly, in these animals miR-200c, which is normally not detectable in the heart, has been shown to be present in Zucker obese rat heart and to activate a compensatory mechanism in order to down-regulate excessive activation of the nutrient sensor kinase S6K1 (Figure 2A) [125]. Another interesting target predicted by bioinformatics algorithms for rno-miR-200c is the mitochondrial enzyme “holocytochrome-c synthetase” (HCCS). HCCS is heme lyase which translocates outside the mitochondria during apoptotic stimuli, suppressing the X-linked inhibitor of apoptosis protein, causing the activation of caspase-3 [126]. Therefore, upregulation of miR-200c can lead to the suppression of HCCS expression that can attenuate cardiomyocyte apoptosis [127].

In this context, the increase of miR-200c supports the notion that in Zucker obese rats compensatory/adaptive mechanisms are activated to attenuate the progression of obesity-induced heart disease, via upregulation of miR-200c.

Mitochondrial dysfunction is known to play a key role also in cardiac abnormalities associated with the type 1 diabetic heart [50,113,128]. In a recent study, an analysis of miRNAs upregulated in diabetic mice heart compared to control was performed; among these miR-200c and miR-141 were the most upregulated [129]. The authors show that miR-141 targets the inner mitochondrial membrane phosphate transporter, “solute carrier family 25 member 3” (Slc25a3), which provides inorganic phosphate to the mitochondrial matrix and is essential for ATP production. Accordingly, Slc25a3 has been shown to be significantly decreased in expression during a type 1 diabetic insult (Figure 2A) [130]. Moreover, Slc25a3 decrease has been shown to exert deleterious effects on ATP production and cell viability [131]. Together, all the evidence indicates an important role of miR-200 family in mitochondrial responses involved in cardiac diseases associated to diabetes and obesity. Given the relevance of miR-200s for vascular cells in this pathological context, it is possible to speculate that similar mechanisms may be present in EC and/or VSMCs as well.

## 5. Challenges and Future Directions

All the evidence mentioned above indicates that miRNAs play a major role in regulating oxidative stress responses in many vascular diseases. Insights into our understanding of these molecular mechanisms and the discovery of novel miRNAs and their protein targets involved in oxidative stress resistance or modulation are of paramount importance. However, data accumulated so far already suggests a new challenging miRNA-based therapeutic strategy for vascular diseases caused by oxidative stress.

MiRNA represents attractive therapeutic targets since they can be easily synthesised both in their sense and antisense (inhibitory) orientation, attached to biomaterials or surgery devices, and used in nanotechnology. Many concerns must be resolved in order to obtain a clinically utilizable strategy for miRNA-based targeted therapy for cardiovascular diseases, including the optimal chemistry and delivery systems for their efficient manipulation as therapeutics. However, it is noteworthy that miRNAs were first shown to function in mammals less than a decade ago, and that the possibility of miRNA manipulation *in vivo* to regulate disease-related processes is already becoming a feasible future therapeutic approach.

Different studies have already taken advantage of miRNA modulation in an attempt to cure vascular complications. It is well known that diabetes accelerates heart, brain, and lower extremities atherosclerotic artery disease [111]. Both animal models and clinical studies show that diabetes impairs cell mobilization from bone marrow (BM), and as a consequence, both the release and the integrity of circulating stem cells is altered [132,133]. Specifically, BM–derived proangiogenic cells (PACs), previously known as early endothelial progenitor cells (EPCs) are implicated in both native and therapeutically guided angiogenesis, which is a novel strategy to support post-ischemic blood flow recovery, wound closure, and tissue regeneration [134]. Recently, it has been shown that critical limb ischemia (CLI) increases miR-15a and miR-16 levels in (PACs) affecting their survival and migration [107]. *Ex vivo*–manipulation of PACs with anti–miR-15a/16 improves the post-ischemic recovery of mice following limb ischemia, indicating that this could be a potential therapeutic strategy to empower PACs before autologous transplantation in patients with CLI [107].

A different study, profiling inbred mouse strains, has shown that a mouse strain displaying higher basal levels of miR-93 and also a stronger induction of this miRNA in response to ischemia shows a better perfusion recovery from hindlimb ischemia [135]. These data indicate that miR-93 may be a potential target for pharmacological modulation to promote angiogenesis in ischemic tissue.

The feasibility of using miRNAs as therapeutic tools is confirmed by the fact that a phase 2a clinical trial adopting an anti-miRNA has been completed successfully (NCT01200420) [136]. In this case an LNA-based anti-miRNA, targeting the liver specific miR-122, has been developed for hepatitis C therapy. This trial showed that the adopted LNA-anti-miRNA is safe and well tolerated; neither dose-limiting toxicities nor discontinuation due to adverse events were reported by patients. Moreover, LNA treatment is associated with dose-dependent, sustained reductions in hepatitis C RNA.

Thus, miRNA targeting offers a very powerful tool through which a protein or a pathway could be finely modulated. However, it must be stressed that possible drawbacks in miRNA therapeutic use could arise since a single miRNA regulates many biological processes, rather than switching a single gene off or on. Therefore, a detailed understanding of the molecular pathways regulated by the miRNA of interest in the specific pathological context must be obtained before moving to patient treatment.

## Figures and Tables

**Table 1:** Relevant miRNAs in oxidative stress response.

ROS source/pathology	miRNAs upregulated	Tissue/organ	Source	Target	Functions	References
H_2_O_2_	miR-200c miR-141 miR-200a miR-200b miR-429	endothelium, myoblasts	Human	ZEB1	apoptosis, senescence	[22]
H_2_O_2_	miR-200c miR-141 miR-200a miR-200b miR-429	ovarian adenocarcinomas	Human	p38α	ROS accumulation; improved response to chemotherapy	[25]
H_2_O_2_	miR-200c	primary hippocampal neurons	Mouse	Unknown	Unknown	[26]
chronic H_2_O_2_ treatment	miR-200c	trabecular meshwork cells	Human	Unknown	senescence	[27]
t-BHP	miR-200c miR-141	auditory cells	Mouse	Unknown	Unknown	[28]
Obesity	miR-200c miR-141	Heart	Rat	S6K1	compensatory/adaptive mechanisms	[29]
Diabetes	miR-200c miR-141	Heart	Mouse	Slc25a3	dysregulation ATP production; cell death	[30]
Diabetes	miR-200c miR-200b miR-429	VSMCs	Mouse	ZEB1	inflammation	[31]
NO	miR-200c miR-200a miR-200b miR-429	mES	Mouse	ZEB2	mesendoderm and cardiovascular differentiation	[32]
Hypoxia/ROS	miR-210	ASCs	Human	PTPN2	proliferation, migration	[33]
Hypoxia	miR-210	ECs, breast and colon cancer cells	Human	ISCU1/2	mitochondrial respiration	[34–36]
Hypoxia	miR-210	breast cancer cells	Human	TfR1	mitochondrial respiration; proliferation	[37]
Hypoxia	miR-210	H9c2 Cardiomyocytes	Mouse	FECH	heme biosynthesis; Iron homeostasis	[38]
Hypoxia	miR-210	colon, breast, esophageal cancer cells	Human	COX10	mitochondrial respiration; ROS production	[39]
Hypoxia	miR-210	lung cancer cells	Human	SDHD	mitochondrial respiration; proliferation	[40]
Hypoxia	miR-210	ovarian cancer cells	Human	NDUFA4	mitochondrial respiration	[41]
Diabetes	miR-125	VSMCs	Mouse	Suv39h1	inflammation	[42]
Obesity	miR-27	Adipose tissue	Mouse	PPARγ, C/EBPα	inflammation	[43]
H_2_O_2_	miR-21	VSMCs	Rat	PDCD4	apoptosis protection	[44]
Coronary artery disease	miR-21	APCs	Human	SOD-2 SPRY-1	ROS production; APC migratory defects	[45]
Atherosclerosis	miR-217	Atherosclerotic plaques	Human	SIRT-1	endothelial dysfunction	[46]
Myocardial infarction	miR-34	BMCs	Human	SIRT-1	apoptosis	[47]
Mitochondrial dysfunction	miR-23a/b	B lymphoma, prostate cancer cells	Human	Mitochondrial GLS	ROS production	[48]
Mitochondrial dysfunction	miR-15 family, miR-424	Cardiomyocytes	Rat	Arl2	ATP reduction	[49]
	downregulated:					
Obesity	miR-155 miR-183 miR-872	Adipose tissue	Rat	HO-1	inflammation, oxidative damage, apoptosis	[50]
Hypoxic preconditioning	miR-199a	Cardiomyocytes	Rat	SIRT-1	apoptosis protection	[51]
H_2_O_2_	miR-23a/b	Retinal pigment epithelial cells	Human	Fas	apoptosis	[52]
